# Supplementary material for: Prevalence of detectable viral load and its associated factors among adult patients receiving ART in Choma District, Zambia
Source: PLoS One. 2025 Apr 3;20(4):e0320571. doi: 10.1371/journal.pone.0320571 (PMC11967971; doi:10.1371/journal.pone.0320571)
Supplement: Attachment — Submitted filename: PONE-D-24-18373_Review Comments.pdf [file pone.0320571.s002.pdf]

Title: Prevalence and factors associated with detectable viral load among adult patients receiving ART in Choma District, Zambia

General comments:

The study estimated the proportion of detectable viral load described associated factors among adults living with HIV receiving antiretroviral therapy (ART) in one of Zambia's 116 districts. While the study is important the narrow geographic scope makes generalization of the study findings to the rest of the country and other resource limited settings unlikely. Nonetheless, the paper has important lessons critical to optimizing care for people living with HIV in this high burden districts. The paper contains terminology that is currently discouraged such as "HIV-infected population" Line 8; infected persons (line 31). These can be rewritten in acceptable terminology following UNAIDS guidelines.

While the study is well conducted and includes data from several large facilities, there are factual and grammatic errors through out the paper. The paper can be made more concise by shortening the background, results, and discussion sections.

A few specific comments to improve the paper are below.

Abstract:

The abstract is beautifully written, but the conclusion could be strengthened by mentioning key factors associated with detectable viremia. The second sentence in the conclusion attempts to do so. In that case part of the first sentence which reads "and were significantly associated with sociodemographic and clinical factors" can be deleted or revised to just state two or three factors that are associated with detectable VL.

Background:

While the background section is comprehensive, the flow can be improved and can be shortened to what is relevant for this article. The section Line 29-42 requires revision and shortening. See more specifics below.

Line 19-24: is too long, quotes several sources but has only one reference. I can be broken into two sentences.

Line 27-28 "for the line that reads "……for females and about 20% of HIV-positive males aged between 24-29 years are 28 not initiated on ART" is there a comparable number for females that should be quoted?

Line 29-30, consider revising the sentence to highlight the gaps rather than stating that "77% of adults and 57% of children" is an improvement unless is over a range.

Line 31-32; requires rewriting to be scientifically accurate and probably mention morbidity and mortality.

Line 32-33; consider revising and including or referencing U=U

Either here or in the methods section, help the reader understand why you chose to study Choma district. Is it that it is a high burden district? Did national data highlight gaps? Does it have challenging terrain?

Methods:

For clarity include a sentence if people with detectable viremia were contacted and interviewed or data were just abstracted from their medical records. The paper states (line 80) that social demographic data were collected but not how.

Proposed revisions:

Line 57 needs a citation/reference.

Line 68: instead of antiretroviral therapy use “ART”

Results:

In general, too many results that are clear in the tables are also written out in sentences and comparisons are not clear. Please reference the table and only highlight significant differences based on the objectives of the study. Paragraph 127-145 can be improved by referring to the relevant table.

All other sections can be similarly rewritten which will make the paper clearer and concise.

Line 112, put K1000 in context by quoting a currency most readers are familiar with such as US$

Line 115 not clear “Only 7 (1.6%) contracted the virus perinatal” correct grammar. But how was this determined?

Line 117-119” The sentence “In terms of nucleoside reverse transcriptase inhibitors (NRTIs), the majority of the clients were on tenofovir disoproxil fumarate/lamivudine (TDF/3TC) (98%) followed by 1.6% zidovudine (AZT)/3TC” Instead of “followed”, use “and”

Line 120-121: please clarify if this is any CD4 or it was a baseline CD4. Also clarify in the methods when CD4 tests are conducted.

Line 121: replace “prevalence” with “proportion.”

Line 123. Not clear what “ordinary counselling service mean.

Line 128. Grammatically not correct or some words are missing. Delete “of them.” Compared to those who did not have what?

Line 146-147 (and all other tables) should include a time horizon. Consider refining the table heading e.g., Prevalence and factors associated with detectable viremia, ……. YYYY-YYYY

Discussion:

Consider stating the key findings as the opening statement. E.G. In Choma District, we found that xxx …. In line with the objectives. The discussion of the results should come in the second paragraph.

Line 237 “~~indicating that 89.7% of the population had undetectable viral load~~ within 12 months of ART initiation” can be deleted.

The discussion is too long and could be shortened by focusing on discussing key findings and putting them in context.

Conclusion:

Consider adding more to the implications.

Line 367: not correct or not clear what the author means "the study demonstrated attainment of"…UNAIDS is mistyped.

373: references instead of reference
