# Supplementary material for: Prevalence of detectable viral load and its associated factors among adult patients receiving ART in Choma District, Zambia
Source: PLoS One. 2025 Apr 3;20(4):e0320571. doi: 10.1371/journal.pone.0320571 (PMC11967971; doi:10.1371/journal.pone.0320571)
Supplement: Attachment — Submitted filename: Response to Reviewers .docx [file pone.0320571.s004.docx]

**Response to Reviewers**

| **Comments** | **Response** |
| --- | --- |
| 1. Data availability – stated “some restrictions will apply” please explain the level of restrictions and how the data can be accessed. | Thank you.  In earlier submission the data had restrictions, however after consideration and appreciating the importance of the data in validation of the manuscript, the data has been uploaded on separate file (S1 minimal dataset) |
| 2. Title: Prevalence and factors associated with detectable viral load among adult patients receiving ART in Choma District, Zambia. “Prevalence” is confusing in the title, is it referring to prevalence of factors or prevalence of detectable VL? Please make it clear, for example “Prevalence of Detectable VL and Associated Factors among Adult Patients Receiving ART in Choma District, Zambia”. | Well noted thank you. The title was revised and shortened accordingly. |
| 3.  Introduction: Good epidemiologic context provided.However, no reference made to factors influencing VL suppression. Consider restructuring the section using 95:95:95 cascade and about 50% of the section should focus on factors influencing VL suppression, including gaps in Zambia and study region. | Thank you, the revision has been made to the introduction. |
| 4. Methods:    - Study design: is it primary data collection or secondary data analysis?    - Selection and sampling: 448 clients were selected. Please describe sampling criteria, method and sample size estimation. Also describe accurately “selection based on proportional to size”.    - Data collection and storage: was the data collected using paper-based questionnaire or electronically? What are the variables of interest and their definition? | Thank you, the section has been revised to and addresses all the concerns reviewers highlighted. |
| 5. Results: not structured and presented without keeping the study objectives in mind. Of course, the study objectives were not well defined in the methods, and this may be the reason for the long and unstructured results section. The section is very lengthy and difficult to follow. No need to mention all findings. Focus on major findings that are elated to the objectives. Try to make the text for this section like maximum of 1 page. The tables should be self-explanatory. The tables are also too long and confusing. Focus on key findings and avoid repetition.    - Table 1: should be “Sociodemographic characteristics and VL suppression of participants from selected health facilities in Choma, Zambia”. The table should end at “perinatal HIV” because the variables that follow are not sociodemographic and should be moved to another table.    - Table 2: Please avoid repetition. Harmonize table 1 and 2 based on the comment above. Make Table 2 to focus on “VL suppression by site and clinical characteristics”    - Tables 3 and 4: please harmonize these tables into 1 table and be concise by focusing on the primary intention of the table. | Well noted, Thank you. The revisions have been made accordingly |
| 6. Discussion: too long. Please be concise and focus on key findings relating to the study objectives. 1.5 pages should be good for well-structured discussions. | Thank you, the revision has been made accordingly. |
| 7. Acknowledgement: though the authors mentioned that no financial assistance received for this study, however it will be appropriate to acknowledge the program that resulted in the data used in the study. Additionally, the 7 hospitals and the clients should be acknowledged. | Thank you. The acknowledgments were revised. |
| 8. References: please review all the references and ensure they align with Plosone citation guidelines. | Thank you, this is well noted. The revision has been made according to provided guidelines. |
| 9. Please ensure that your manuscript meets PLOS ONE's style requirements, including those for file naming | Thank you. This has been done accordingly. |
| 10. Reviewer #1:   1. Among the participants who received enhanced adherence counselling, a significant 140 proportion of them had detectable VL compared with the ones who did not have detectable VL, 141 32.6% vs. 0.5%, p <0.001 (row 140 and 141, this sentence is standalone and not explanatory) 2. Row 142. A higher prevalence of the clients aged between 16 and 24 years had detectable VL compared with those without detectable VL, 15.2% vs. 6.2% ( i suggest it can be a higher percentage or proportion rather than prevalence) | Thank you, well noted. The revision has been made accordingly to make the information easier to understand. |
| 11. Reviewer #2: This paper addressed a very important topic. I have provided some detained comments in the attached documents to help improve the flow and readability. The authors can consider shortening the paper, restructuring the background and results section and discussing the key findings. | The comments in the attached document were well appreciated. Thank The revision has been made. |
| 12. Reviewer #3: Short title has a minor grammatical error, remove 'of'. The inclusion and exclusion criteria is missing. There is need to show the age of consent and who needs assent. The Consent form was only in English, looking the population ,there was need to have a translated version to native language. Researcher may need to explain how the questionnaire was administered. The data was not available in the manuscript | Thank you so much. The title has been shortened accordingly and the concerns addressed in the methods section. |
